# ProNGF derived from rat sciatic nerves downregulates neurite elongation and axon specification in PC12 cells

**DOI:** 10.3389/fncel.2015.00364

**Published:** 2015-09-15

**Authors:** Anna Sofía Trigos, Marines Longart, Lisbeth García, Cecilia Castillo, Patricia Forsyth, Rafael Medina

**Affiliations:** ^1^Unidad de Neurociencias, Instituto de Estudios Avanzados (IDEA)Caracas, Venezuela; ^2^Departamento de Biología Celular, Universidad Simón Bolívar (USB)Caracas, Venezuela

**Keywords:** neuronal differentiation, proNGF, PC12 cells, axons, sortilin, sodium channels, conditioned medium

## Abstract

Several reports have shown that a sciatic nerve conditioned media (CM) causes neuronal-like differentiation in PC12 cells. This differentiation is featured by neurite outgrowth, which are exclusively dendrites, without axon or sodium current induction. In previous studies, our group reported that the CM supplemented with a generic inhibitor for tyrosine kinase receptors (k252a) enhanced the CM-induced morphological differentiation upregulating neurite outgrowth, axonal formation and sodium current elicitation. Sodium currents were also induced by depletion of endogenous precursor of nerve growth factorr (proNGF) from the CM (pNGFd-CM). Given that sodium currents, neurite outgrowth and axon specification are important features of neuronal differentiation, in the current manuscript, first we investigated if proNGF was hindering the full PC12 cell neuronal-like differentiation. Second, we studied the effects of exogenous wild type (pNGF*wt*) and mutated (pNGF*mut*) proNGF isoforms over sodium currents and whether or not their addition to the pNGFd-CM would prevent sodium current elicitation. Third, we investigated if proNGF was exerting its negative regulation through the sortilin receptor, and for this, the proNGF action was blocked with neurotensin (NT), a factor known to compete with proNGF for sortilin. Thereby, here we show that pNGFd-CM enhanced cell differentiation, cell proportion with long neurites, total neurite length, induced axonal formation and sodium current elicitation. Interestingly, treatment of PC12 cells with wild type or mutated proNGF isoforms elicited sodium currents. Supplementing pNGFd-CM with pNGF*mut* reduced 35% the sodium currents. On the other hand, pNGFd-CM+pNGF*wt* induced larger sodium currents than pNGFd-CM. Finally, treatments with CM supplemented with NT showed that sortilin was mediating proNGF negative regulation, since its blocking induced similar effects than the pNGFd-CM treatment. Altogether, our results suggest that proNGF within the CM, is one of the main inhibitors of full neuronal differentiation, acting through sortilin receptor.

## Introduction

Early reports have shown that explanted sciatic nerves promote axonal regrowth in the injured Central Nervous System (CNS; Benfey and Aguayo, 1982; Sandrock and Matthew, 1987). Importantly, a sciatic nerve conditioned media (CM), obtained from the degeneration of rat sciatic nerves in culture, contains neurotrophic factors that promote differentiation towards a neuronal-like morphology in PC12 cells (Villegas et al., 1995). However, other studies have demonstrated that PC12 cell treatment with this media is unable to generate sodium currents (Castillo et al., 2001), which is an important requisite for cell differentiation and polarization (Wada, 2006). Additionally, voltage-dependent sodium channels are necessary for clustering of key proteins, such as cell adhesion proteins (ankyrin G, NrCAM and neurofascin), which help in the structural stabilization of the axon initial segment (Xu and Shrager, 2005).

Previous reports from our group reported that PC12 cells treated with CM only developed dendrites (Longart et al., 2009). This, added to the absence of sodium currents and axons in cells treated with the CM, suggested that this media is constituted by factors that can either promote or limit its neurotrophic capability. Partial characterizations of the CM have shown that it contains neuregulin-1 and glypican-1, which were involved in the CM neuritogenic activity. At the same time the actions of some neurotrophic factors and mature neurotrophins i.e., NGF, brain-derived neurotrophic factor (BDNF), Ciliary neurotrophic factor (CNTF) and fibroblast growth factors were ruled out (Villegas et al., 1995, 2000; Malavé et al., 2003). Many studies have demonstrated the role of NGF in neuronal survival and differentiation (Huang and Reichardt, 2001; Zhou et al., 2004). The signals mediated by NGF induce rapid axon outgrowth, through a mechanism dependent of a tightly regulated and localized activation of phosphatidylinositol 3-kinase (PI3K) in the growth cones, involving changes in the axonal cytoskeleton (Zhou et al., 2004). Nevertheless, little is known about the role of proneurotrophins, like proNGF, in cell differentiation and how these factors can regulate crucial aspects of neuronal differentiation, including sodium current generation and axon development.

ProNGF has been found in a wide variety of tissues, with high expression in mouse, rat and human CNS, concomitant with low levels of mature NGF (Fahnestock et al., 2001); which suggest that proNGF might play important physiological functions in these tissues, beyond the classical role as a precursor to produce mature NGF or as a pro-apoptotic molecule (Lee et al., 2001; Beattie et al., 2002; Domeniconi et al., 2007). The seemingly conflicting reports, about the biological activity of proNGF, suggest the idea of multiple roles for this molecule. Indeed, it was shown that proNGF promotes neurite outgrowth in a subset of NGF-dependent neurons (superior cervical ganglion, SCG) by inducing a diffuse growth during the developmental stage when axons are branching in their target tissues. This effect was dependent on the p75 receptor and did not require receptor tyrosine kinase (TrK) activation (Howard et al., 2013). In other studies, SCG neurons, treated with cleavage-resistant (mutated proNGF) or wild type proNGF isoforms, resulted in neuronal survival and promotion of neurite outgrowth in SCG and PC12 cells (Fahnestock et al., 2004). Additionally, proNGF can be either neurothrophic (PC12 cells and sympathetic neurons) or apoptotic (PC12 cells), depending upon relative levels of its receptors (Masoudi et al., 2009). On the other hand, studies of the signaling pathways used by proNGF to exert its neurotrophic role have shown that this proneurotrophin was able to elicit phosphorylation of tropomyosin-related kinase A (TrkA) receptor and extracellular-signal-regulated kinases 1/2 (ERK1/2) in NIH3T3-TrkA and human embryonic kidney 293 cells (Fahnestock et al., 2004; Clewes et al., 2008).

In previous studies, we showed that, supplementing the CM with a generic inhibitor for tyrosine kinase receptors (k252a) caused neuronal-like differentiation with an increase of neurite outgrowth and length, the induction of axon formation and sodium current elicitation. In addition, it was shown that various proNGF isoforms were present in the CM and their removal by immunoprecipitation (pNGFd-CM) caused sodium current elicitation in PC12 cells (Longart et al., 2009). These results were already suggesting that proNGF could be involved in the negative regulation, exerted by the CM, over the neuronal-like differentiation of the PC12 cells. Given the presence of proNGF in the CM and the limitations of this medium to accomplish full morphologycal differentiation, it was very important to further study how this molecule, within the CM environment, would modulate neurite outgrowth and cell polarization.

Consequently, in the current manuscript, we investigated if proNGF in the CM was one of the specific inhibitors of neuronal-like differentiation, neurite outgrowth and axon specification. For these experiments we used the modified CM, where proNGF was immunodepleted (pNGFd-CM). Additionally, considering that pNGFd-CM induces sodium currents, we sought to further investigate the effect of exogenous proNGF isoforms (mutated or wild type) and if reconstitutions of pNGFd-CM with these exogenous isoforms were able to reinstate the CM with its inhibitory functions over sodium currents. Last, given that proNGF and the neuropeptide neurotensin (NT) bind to the sortilin receptor, and NT competes with proNGF for binding to sortilin (Nykjaer et al., 2004; Domeniconi et al., 2007; Al-Shawi et al., 2008), we investigated if sortilin was involved in the negative regulation over the neuronal differentiation exerted by proNGF by supplementing the CM with NT. Our results suggest that proNGF within the CM, is one of the main inhibitors of neuronal differentiation and its neutralization or modulation are necessary to accomplish a completely functional neuronal differentiation.

## Materials and Methods

### Materials

Protein A Sepharose was from GE Healthcare (Buckinghamshire, UK). Rabbit anti-pan NGF (H-20) antibody was from Santa Cruz Biotechnology (Santa Cruz, CA, USA). Human recombinant proNGFs, wild type (pNGF*wt*) and mutated (pNGF*mut*) were from Alomone Labs (Jerusalem, Israel). NT was from Calbiochem (La Jolla, CA, USA). As axonal marker it was used the mouse anti-phosphorylated neurofilament H antibody (SMI-35; Covance, Princeton, NJ, USA) and as dendritic marker, the rabbit anti-MAP2 antibody (Chemicon; Temecula, CA, USA). The secondary antibodies Goat-anti rabbit Alexa Fluor 594 and goat-anti mouse Alexa Fluor 488 were from Invitrogen (Carlsbad, CA, USA). DAPI was from Calbiochem (La Jolla, CA, USA).

### Animals

Sciatic nerves were extracted from Sprague–Dawley adult rats and were used to prepare the CM. Animals were kept and sacrificed following the regulations of the Instituto de Estudios Avanzados (IDEA), in accordance with the National Institutes of Health Guide for the Care and Use of Laboratory Animals (NIH Publications No. 80–23), revised in 1996. Care was taken to use the minimal amount of animals and all precautions were taken to minimize any suffering.

### Preparation of CM Immunodepleted of proNGF Isoforms (pNGFd-CM)

The CM was prepared as previously indicated (Villegas et al., 1995), with some modifications. Briefly, Sprague–Dawley adult rats were used to extract the sciatic nerves and prepare the CM. For this, groups of eight nerves were cultured in 6 ml Dulbecco’s modified eagle medium (DMEM) without sera during 7 days. At day 8, the nerves were transferred to new flasks with fresh serum-free DMEM. CM was collected every 24 h and maintained at −70°C until use. For cell treatments, the media from days 9, 10 and 11 were pooled together and supplemented with 2.5% fetal bovine serum (FBS) and 1.25% horse serum (HS). To prepare pNGFd-CM, we followed the procedure previously reported (Longart et al., 2009). Briefly, aliquots of CM were pre-absorbed with Protein A Sepharose for 1 h, at room temperature with constant agitation. The mixture was spun at 2000 rpm for 4 min. The supernatant was incubated overnight with 4 μg/ml of anti-pan NGF antibody, at 4°C with constant agitation. This antibody recognizes both mature and immature NGF isoforms. Next, the mix was incubated with Protein A Sepharose for 1 h at room temperature and centrifugated at 2000 rpm for 4 min. The Protein A Sepharose pellets, resultant of the immunoprecipitation, were analyzed by Western blot to verify the efficacy of the procedure. Additionally, the efficacy of proNGF removal was measured by the ability of the pNGFd-CM to induce sodium currents. The supernatant, representing the pNGFd-CM, was stored at 4°C until its use.

### Cell Culture and Treatments

PC12 cells were grown in DMEM (Dulbecco Modified Eagle’s Medium; Gibco-Invitrogen, Carlsbad, CA, USA) supplemented with 10% FBS, 5% HS, 2 mM glutamax (Gibco-Invitrogen, Carlsbad, CA, USA) and 1% penicillin–streptomycin (Sigma, St. Louis, MO), in 5% CO_2_ at 37°C. In all experiments, 2 h after seeding, cells were fed with medium containing 2.5% FBS and 1.25% HS. The culture medium was changed every 3 days. For electrophysiological experiments and morphological analysis, cells were plated in 24 or 48-well plates, 3000–6000 cells per well, and cultured with the corresponding treatments. For immunocytochemistry experiments, cells were cultured on 24 well plates treated with Poly-L-lysine (PLL; 10 mg/ml, Sigma).

All peptides were dissolved in DMEM. The effect of proNGF isoforms, wild type (pNGF*wt*) and mutated (pNGF*mut*), over sodium currents was measured by electrophysiological experiments. The proNGF isoforms were tested alone or in combination with pNGFd-CM. Both isoforms were used at 10 and 100 ng/ml. NT was used at 10 and 40 μM considering that, concentrations of 10 μM (Domeniconi et al., 2007) and 40 μM (Nykjaer et al., 2004; Al-Shawi et al., 2008) are enough to block the effect caused by the binding of proNGF to sortilin.

### Morphometric Analyses

Morphometric analyses were carried out on PC12 cells after 10 days of treatment. To define the degree of cell differentiation, two criteria were considered: the largest cell body diameter and the neurite length. Cells with cell body diameters greater than 15 μm and without neurites (or with neurites smaller than 2.5 μm), were considered as differentiated without neurites (DWN). Cells with at least one neurite with length above 12 μm, were considered as differentiated with long neurites (DLN). Cells with neurites between 2.5 and 12 μm, were considered as differentiated with short neurites (DSN). The average total neurite length per cell was obtained by adding the length of all neurites (short and long), divided by the number of cells with neurites, as previously described (Longart et al., 2009; García et al., 2013).

Cells were visualized with a transmitted light microscope Axiovert 100 (Carl Zeiss, Jena, Germany) and a dry objective, 32X/1.4. Images were acquired with a digital camera (Nikon Coolpix 990), using the Nikon View 6 Software (2000). Image analysis and processing were performed using the Image J software (NIH, Bethesda, MD, USA).

### Electrophysiology

Sodium currents were measured at 4–7 days of treatment. Cells were resuspended and plated on cover slips treated with type 1 collagen (Sigma). Measurements were initiated after 2 h of incubation at 37°C in a 5% CO_2_ atmosphere. Whole-cell current recordings were performed according to standard techniques with a patch-clamp amplifier (Axopatch 200A; Molecular Devices, Sunnyvale, CA, USA), using an inverted microscope. Data were acquired with pCLAMP 9.0 software (Molecular Devices). PC12 cells were voltage-clamped with the tight-seal whole-cell patch-clamp method (Hamill et al., 1981) using electrodes with resistance of 2–4 MΩ after being filled with the internal solution. Most capacitive transient currents were cancelled with the electronic circuitry provided by the amplifier and leak subtraction was performed using a standard P/6 protocol. All measurements were performed at 20–21°C. Sodium (Na^+^) currents were recorded at a step potential of −10 mV, steady-state activation was studied using a conventional two-pulse protocol from a holding potential of −120, from −80 to +60 mV (10 mV, 20 ms steps) with repetition interval of 3 s. Only cells that looked differentiated, according to the largest diameter of the cell bodies were used. Cells with visible apparent Na^+^ currents larger than 100 pA were considered to have sodium currents. The external solution consisted of: 120 mM NaCl, 2.8 mM KCl, 20 mM TEA-Cl (tetraethylamonium chloride), 2 mM MgCl_2_, 1 mM CaCl_2_, 10 mM HEPES and 5 mM glucose, and pH was adjusted to 7.2 with 25% NaOH. The internal solution consisted of: 105 mM CsF, 35 mM NaCl, 10 mM EGTA and 10 mM HEPES, adjusted to pH 7.2 with CsOH.

### Immunocytochemistry

PC12 cells were grown on plastic surface (polystyrene) and incubated with each treatment for 13–14 days. All procedures were performed at room temperature, unless otherwise indicated. Cells were fixed with 4% paraformaldehyde (PFA) in PBS for 20 min, washed with PBS and then permeabilized with 0.25% Triton X-100 in PBS for 10 min. Thereafter, the cells were blocked with 10% normal goat serum (NGS) in PBS for 1 h. Cultures were incubated with the primary antibodies SMI-35 (1:5000, mouse) and MAP2 (1:5000, rabbit), diluted in 2% NGS in PBS, overnight at 4°C, followed by incubation with the secondary antibodies, anti-rabbit Alexa Fluor 594 (1:300) and anti-mouse Alexa Flour 488 (1:200), during 1 h. DAPI was used to visualize the nucleus.

The neurites with less than 50% of its length labeled with the axonal marker (SMI-35 antibody) were considered nascent axons; while the neurites with more than 50% of its length labeled with the axonal marker, were considered maturing axons. Cells were observed in an AxioObserver Z.1 fluorescent inverted microscope (Carl Zeiss, Jena, Germany) with a dry objective, 40X/0.6. Images were acquired with a CCD camera (Photometrix Cool SNAP HQ2-Photometri, Tucson, AZ, USA). Management of images was carried out using the Roper Scientific software with an exposure time within the lineal range. Image analysis and processing were performed with the Image J software (NIH, Bethesda, MD, USA). Figures were prepared in Adobe Photoshop CS5 and minor adjustment of brightness and contrast were performed.

### Statistical Analysis

Values are presented as mean ± SEM. For electrophysiological experiment analysis, differences were evaluated using Kruskal-Wallis with Dunn’s multiple comparison post test when nonparametric test were used. For morphometric analysis, differences were evaluated by two-tailed *t*-test and one-way ANOVA with Holm-Sidak’s multiple comparison post test or Kruskal-Wallis with Dunn’s multiple comparison post test (GraphPad Prism 5.0, GraphPad Software, San Diego, CA, USA).

## Results

### Treatment of PC12 Cells with pNGFd-CM Enhanced Cellular Differentiation Inducing Neurite Outgrowth and Axon Specification

**Figure 1:**
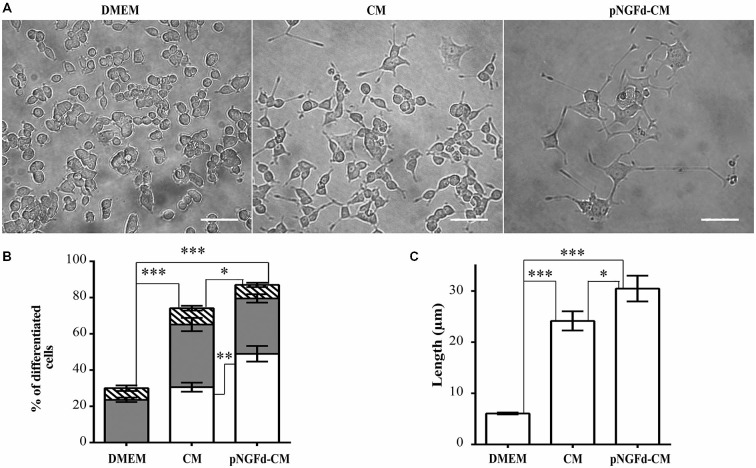
**pNGFd-CM increases neurite outgrowth. (A)** DIC images of PC12 cells treated with DMEM, CM and pNGFd-CM. Scale bars = 50 μm. **(B)** Graph bars show the percentages of cell differentiation under different treatments; parameters measured were: total differentiation (whole bars), differentiated cells with long neurites (white bars), differentiated cells with short neurites (gray bars), and differentiated cells without neurites (hatched bars). For total cell differentiation experiments, treatment with pNGFd-CM induced larger differentiation than CM (*p < 0.05) and DMEM (***p < 0.001) and these two treatments induced larger differentiation than DMEM treatment (***p < 0.001). Regarding the effect of the different treatments on the induction of long neurites, pNGFd-CM, compared to CM, increased the proportion of long neurites in differentiated cells (**p < 0.01; while the proportion of short neurites were similar between these two treatments (*p* > 0.05; two-tailed *t*-test). Similarly, these two treatments did not show differences in the proportion of short neurites in comparison to DMEM. **(C)** Graph bars show the total neurite length in PC12 cells after 10 days of treatment with DMEM, CM and pNGFd-CM. Total neurite length was increased in cells treated with pNGFd-CM, in comparison to CM (*p < 0.05) and these two treatments induced longer neurites than the DMEM (***p < 0.001; One way ANOVA with Holm-Sidak’s multiple comparison test). Graph values represent the mean ± SEM. *N* = 3 independent experiments. Total number of cells: DMEM, *n* = 1569; CM, *n* = 3120; pNGFd-CM: *n* = 1333.

To evaluate the effect of endogenous proNGF, present in the CM, on PC12 cell differentiation, proNGF was immunoprecipitated from the CM (pNGFd-CM). The percentage of differentiated cells treated with pNGFd-CM (87.01 ± 3.76%; *n* = 4) was higher that the observed with native CM (70.61 ± 2.7%; *n* = 4; *p* < 0.05; Figure 1B) and these two treatments induced higher percentage of differentiation in comparison with the control treatment (30.00 ± 2.66%; *p* < 0.001). PC12 cells treated with pNGFd-CM developed neurites which were longer than the ones developed in cells treated with CM alone (Figures 1A,B). The quantification showed that the pNGFd-CM caused an increase in the proportion of cells with long neurites to 48.98 ± 4.31%, in comparison to 29.16 ± 1.84% observed with the CM (*p* < 0.01); while the percentage of cells with short neurites showed no differences among the three treatments (DMEM = 23.59 ± 1.21%, *CM* = 34.58 ± 3.67%, pNGFd-CM = 30.56 ± 2.32%; *p* > 0.05; Figure 1B). Furthermore, the pNGFd-CM treatment induced neurite elongation that were evident with the estimation of total neurite length, which was 30.46 ± 2.52 μm, whilst for the CM the total neurite length was 22.7 ± 1.54 μm (*p* < 0.05; Figure 1C). Pre-treatment of the CM medium with Protein A Sepharose did not cause any effect on the percentages of differentiated cells with long neurites, nor in the neurite length. There were no visual or numerical differences in any of the parameters between the treatments with CM and CM pre-absorbed with Protein A (*data not shown*).

**Figure 2:**
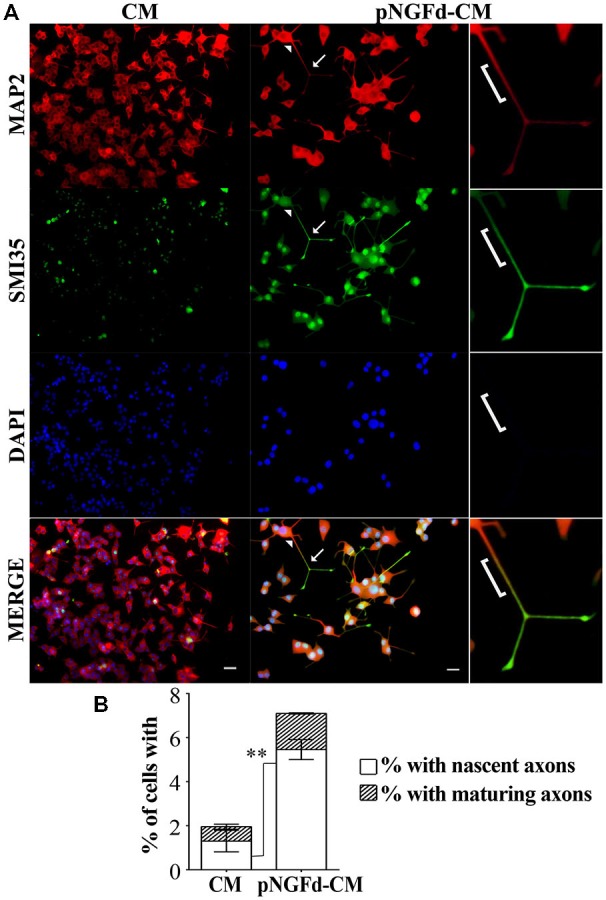
**pNGFd-CM promotes axonal generation and maturation. (A)** Immunofluorescence images of PC12 cells treated with CM and pNGFd-CM for 13–14 days. Cells were double labeled with antibodies for dendrites (MAP 2, red) and axons (SMI35, green). DAPI was used to visualize the nucleus. Arrowhead point to a neurite proximal part, bracket indicates the neurite middle part, expressing the dendritic and the axonal markers and arrows indicate the axons (distal part of the neurites). The insets show enlargement of the cell parts pointed by arrows. Scale bars = 20 μm. **(B)** Graph bars show the percentages of cells with axons (whole bars), nascent axons (white bars) and maturing axons (hatched bars). Graph values represent the mean ± SEM. **p < 0.01 (two-tailed *t*-test). Maturing axons were not different between CM and pNGFd-CM (*p* > 0.05). *N* = 3 independent experiments. Total number of cells: CM, *n* = 466; pNGFd-CM, *n* = 291.

Our group previously demonstrated that immunoprecipitation of proNGF isoforms from the CM was an efficient process, which was shown by Western blot in previous (Longart et al., 2009) and present work (data not shown). Additionally, in our previous (Longart et al., 2009) and present work, we demonstrated that pNGFd-CM promoted the generation of sodium currents. In consequence, this was another way to directly test the efficiency of the immunoprecipitation procedure. On the other hand, there is a relation between sodium channel expression and neuronal polarization (Wada, 2006). Therefore, immunofluorescence assays were carried out to evaluate if treatment of PC12 cells with pNGFd-CM induced axonal formation. As predicted, pNGFd-CM, induced axonal formation and outgrowth, while it was confirmed that the CM was only able to produce dendrites. This can be clearly observed in Figure 2, where with the CM treatment the axonal marker did not show any specific labeling (Figure 2A, left panels). It is important to note that we observed that both, the dendritic and the axonal markers, were expressed in the longer processes that would become axons, following a gradation. As shown in Figure 2A (right panel), the arrowhead points to the proximal part of a neurite, there the labeling looked more intense for the dendritic marker. Towards the middle part of the processes, this dendritic marker started to disappear and the axonal marker started to show, which is indicated by a yellow-orange color in the merged panel (bracket). Importantly, the labeling for the axonal marker looked more intense and defined at the axon endings, (Figure 2A, arrows). These results show a pre-polarization stage in the neurites that will become axons, in PC12 cells. Another observation is that the pNGFd-CM seemed to induce a major degree of neuronal-like differentiation, with larger cell bodies and longer neurites, in comparison to the CM.

A more detailed study of axonal formation in cells treated with native CM showed that this medium generated a low percentage of cells with axons (1.96 ± 0.60%). These axons were almost exclusively nascent axons (1.32 ± 0.49%) and only few cells showed maturing axons (0.65 ± 0.10%; Figure 2B). In contrast to the low number of axons observed with CM, the cell treatment with pNGFd-CM produced an increase of neurites labeled with the axonal marker (Figure 2B; 7.10 ± 0.42%, *p* < 0.05) and a higher percentage of cells with nascent axons (5.46 ± 0.45%, *p* < 0.01). Finally, the percentage of cells with maturing axons (1.64 ± 0.03%) in the pNGFd-CM treatment tended to increase over the percentage of cells treated with native CM; however, these differences were not significant (*p* > 0.05). These results indicate that the absence of proNGF in the CM, additional to axonal specification, might promote axonal maturation.

### Exogenous proNGF Isoforms Alone or in Combination with pNGFd-CM Regulate Sodium Current Elicitation

**Figure 3:**
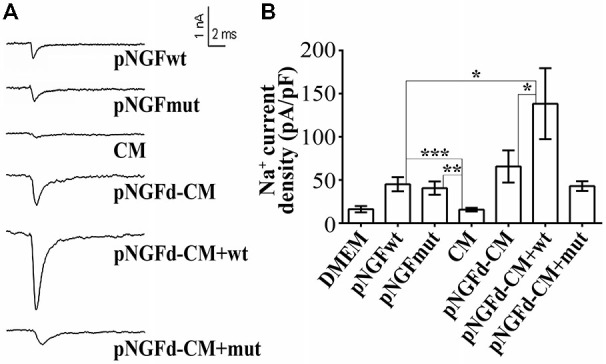
**Effect of exogenous wild type (pNGFwt) and mutated (pNGFmut) proNGF isoforms tested alone or in combination with pNGFd-CM, over sodium current induction. (A)** Representative sodium current traces with the different treatments. **(B)** Graph bars show sodium channel densities after 4–7 days of treatment. Sodium current densities elicited by treatment with *wt* and *mut* proNGF isoforms were larger than the induced by CM. Bars represent the mean ± SEM. *p < 0.05, **p < 0.01, ***p < 0.001 (non parametric *t*-test). Number of cells, from the total, with sodium currents: DMEM, *n* = 10/26 (38.46%); DMEM+pNGF*wt*, *n* = 8/10 (80%); DMEM+ pNGF*mut*, *n* = 9/11 (81.82%); CM, *n* = 13/25 (52%); pNGFd-CM, *n* = 12/15 (80.00%); pNGFd-CM+ pNGF*wt*, *n* = 7/7 (100%); pNGFd-CM+ pNGF*mut*, *n* = 20/21 (95.24%).

Previous findings from our laboratory have demonstrated that the number of cells with sodium currents, as well as their sodium channel densities, were increased by proNGF removal from the CM, suggesting an inhibitory role of this molecule over these parameters (Longart et al., 2009). In this sense, we investigated the effect of exogenous proNGF isoforms and studied if supplementing the pNGFd-CM with these isoforms would reinstate the CM with the original inhibitory properties over the sodium currents. These experiments were performed using a wild type isoform (pNGF*wt*) and a mutated isoform (pNGF*mut*), which cannot be processed to its mature form by the protease Furin. As expected, PC12 stimulated with pNGFd-CM, showed an elicitation of sodium currents, while no sodium currents were observed with CM stimulation (Figures 3A,B). The proNGF isoforms, pNGF*wt* and pNGF*mut*, increased sodium current densities and their values were statistically different from the observed with DMEM or CM (pNGF*wt* 45.1 ± 8.15 pA/pF, *p* < 0.001 and pNGF*mut* 40.6 ± 7.7 pA/pF, *p* < 0.01). Interestingly, stimulation with pNGFd-CM supplemented with pNGF*mut* induced sodium current densities that were 35% smaller (42.89 ± 5.6 pA/pF) than those observed with pNGF-CM (65.7 ± 18 pA/pF) but were not significantly different. Surprisingly, when cells were stimulated with pNGFd-CM supplemented with pNGF*wt* isoform, currents were larger (138.3 ± 41 pA/pF, *p* < 0.05) than those obtained with pNGFd-CM or pNGFd-CM supplemented with pNGF*mut* (Figures 3A,B). Since there was not numerical difference between 10 or 100 ng/ml treatments with commercial proNGF isoforms (wt and mut), measurements with both concentrations were pooled together. Percentage of cells expressing sodium currents were: 38.46% (DMEM), 52.00% (CM), 80.00% (pNGF*wt*), 81.82% (pNGF*mut*), 80.00% (pNGFd-CM), 100% (pNGFd-CM+pNGF*wt*) and 95.45% (pNGFd-CM+pNGF*mut*). Sodium current density values were: DMEM = 16.19 ± 3.65 pA/pF and *CM* = 15.67 ± 2.14 pA/pF. In general, it was observed a higher percentage of cells expressing sodium currents in treatments with the highest current densities, and a lower percentage of cells expressing sodium currents in treatments with the lowest current densities.

### ProNGF Downregulates Neuronal-Like Differentiation of PC12 Cells Through the Sortilin Receptor

**Figure 4:**
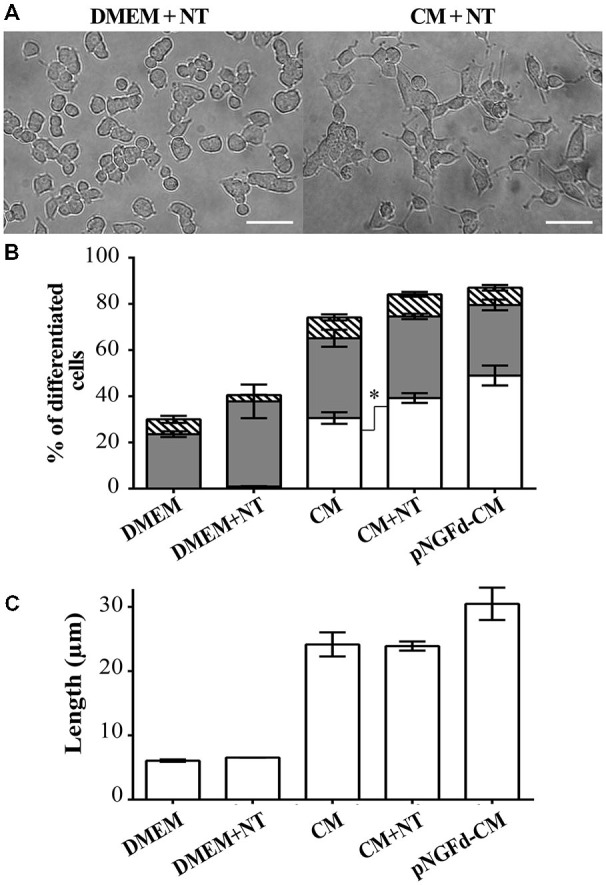
**Blockage of proNGF binding to sortilin with Neurotensin (NT) induces neurite outgrowth and elongation. (A)** DIC images of PC12 cells treated with DMEM+NT and CM+NT. Scale bars = 50 μm. **(B,C)** show results from PC12 cells after 10 days of treatment with: DMEM, DMEM+NT, CM, CM+NT and pNGFd-CM. **(B)** Graph bars show the percentage of cell differentiation under the indicated treatments, and the measured parameters were: total differentiation (whole bars), differentiated cells with long neurites (white bars), short neurites (gray bars) and without neurites (hatched bars). Treatment with CM+NT increased the percentage of cells with long neurites, respect to CM alone, without affecting the other parameters. DMEM and DMEM+NT did not show significant differences in the analyzed parameters. **(C)** Graph bars show that treatment with CM+NT did not increase the total length of neurites (TLN), respect to the CM alone. Bars represent the mean ± SEM. *p < 0.05 (two-tailed *t*-test). *N* = 3 independent experiments. Total number of cells: DMEM, *n* = 1569; DMEM+NT, *n* = 653; CM, *n* = 3120; CM+NT, *n* = 1741.

Given that NT can compete with proNGF for the binding to the sortilin receptor (Nykjaer et al., 2004), we incubated PC12 cells with CM supplemented with NT in an attempt to inhibit proNGF action by blocking this receptor. If sortilin was mediating the proNGF effect, thus the addition of NT to the CM should mimic the effect of depleting proNGF from the CM. Consequently, treatment with CM supplemented with NT, increased overall cell differentiation (84.16 ± 2.46%) in comparison to native CM (70.61 ± 2.70%; *p* ±.0.05; Figures 4A,B). In cells treated with CM+NT, the percentage of differentiated cells with long neurites also increased (39.24 ± 2.10%), in comparison to CM (30.56 ± 2.5%, *p* ± 0.05). However, the total neurite length remained similar to the one observed in CM (CM+NT: 23.89 ± 0.71 μm, CM: 21.10 ± 2.06 μm; Figure 4C). Control experiments, where PC12 cells were treated with DMEM and DMEM+NT showed no statistical differences (*p* > 0.05), for the parameters of total cell differentiation (DMEM = 30.00 ± 2.66%; DMEM+NT = 40.55 ± 7.50%) and differentiated cells with short neurites (DMEM = 23.60 ± 1.21%; DMEM+NT = 36.85 ± 7.28%).

**Figure 5:**
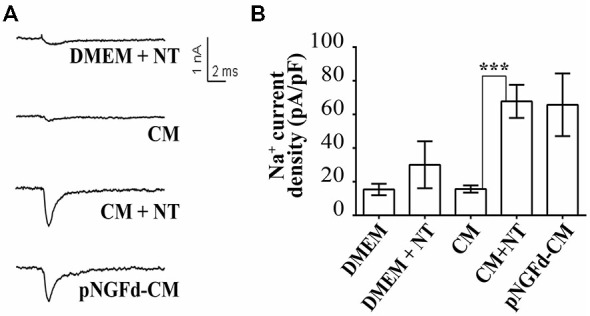
**Blockage of proNGF binding to sortilin with NT promotes induction of sodium currents in PC12 cells. (A)** Representative sodium current traces with the different treatments. **(B)** Graph bars show sodium channel densities after 4–7 days of treatment. CM+NT and pNGFd-CM treatments increased sodium current in a similar fashion but at the same time very different from the CM. Bars represent mean ± SEM. ***p < 0.001 (Kruskal-Wallis with Dunn’s comparison multiple test). Number of cells, from the total, with sodium currents: DMEM, *n* = 10/26 (38.46%); CM, *n* = 13/25 (52%); pNGFd-CM, *n* = 12/15 (80.00%); DMEM+NT, *n* = 5/9 (55.56%); CM+NT, *n* = 23/26 (88.46%). Values for DMEM, CM and pNGFd-CM are the same shown in Figure 3 and are shown also here to facilitate comparisons.

**Figure 6:**
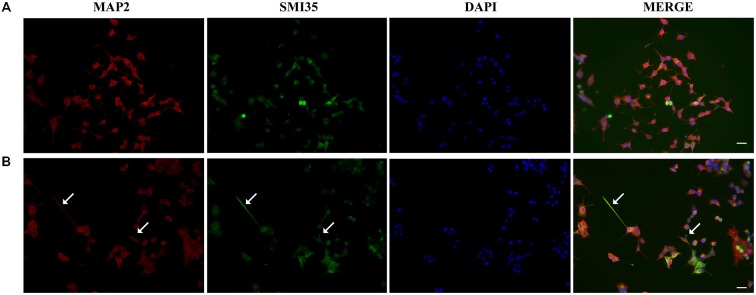
**Blockage of proNGF binding to sortilin with NT promotes axon formation.** Panels show immunofluorescence images of PC12 cells treated with **(A)** CM and **(B)** CM+NT for 13–14 days, double labeled with antibodies that recognize dendrites (MAP2, red) and axons (SMI35, green), DAPI was used to visualize the nucleus. Arrows point the axons. Scale bars = 20 μm.

Next, we studied the effect of CM+NT over sodium currents and found that this treatment also promoted their induction (Figures 5A,B). Indeed, the sodium current densities elicited by the CM+NT treatment (67.77 ± 14.14 pA/pF; Figure 5B) were similar to those induced by pNGFd-CM treatment (65.7 ± 18.00 pA/pF; Figure 5B). CM+NT treatment (67.77 ± 14.14 pA/pF) was different from CM, (15.67 ± 2.14 pA/pF, *p* < 0.001) and from DMEM+NT (30.1 ± 14.00 pA/pF, *p* < 0.01). Percentage of cells expressing sodium currents were: 55.56% (DMEM+NT) and 88.46% (CM+NT). Values for the percentages of cells with sodium currents and for the current densities DMEM, CM, and pNGFd-CM) are the same values for the results shown in Figure 3. Since there were no differences in the morphological parameters or in sodium current elicitation, with the two tested concentrations of NT (10 and 40 μM), these results were pooled together. Finally, using immunofluorescence we showed that the CM+NT treatment (Figure 6B), like the pNGFd-CM treatment, was able to induce axonal formation. Note the absence of the axonal marker in the CM treatment (Figure 6A).

## Discussion

This work demonstrates that proNGF in the CM functions as a negative regulator of the full neuronal-like differentiation. This conclusion is supported by two main findings of the present work and by previous finding showing the proNGF negative regulation over sodium current induction. In the present work, first we showed that removal of proNGF from the CM, enhanced neurite outgrowth and elongation and started axonal formation in PC12 cells. Second, the negative effect over sodium current induction and axonal formation is exerted by proNGF through the sortilin receptor. The role of proNGF on axonal specification and the involvement of sortilin on axonal specification and sodium current induction are important findings described here for the first time. It is important to mention that our current results support roles for proNGF on neurite outgrowth and specification that represent a counterparty of its classical roles as a precursor to produce mature NGF or as a cell death promoter (Lee et al., 2001; Beattie et al., 2002; Domeniconi et al., 2007). The fact that the CM induces morphological differentiation raises several alternatives for the proNGF function on PC12 cell differentiation. It is possible that proNGF might initiate neurite outgrowth, although limited, and render an incomplete morphological and functional differentiation. In support of this, previous findings from our group demonstrated that specific inhibition of TrkA and the p75^NTR^ receptors reduced the neurite length in CM-treated cells, in comparison with cells treated only with CM (Longart et al., 2009). Considering that proNGF can interact with sortilin, p75^NTR^ (Nakamura et al., 2007) and Trk A (Masoudi et al., 2009), it was logical to consider the involvement of p75^NTR^ or TrkA receptors, over the initial and limited neuritogenic effect, which would be only represented by short neurites or dendrites on PC12, caused by the proNGF present in the native CM. At the same time, the CM might provide with another mechanism through which the binding of proNGF to sortilin would avoid the induction of axonal formation and of sodium currents. Thus, after the initial differentiation, other molecules are needed to further induce neurite elongation, to produce axons and sodium currents. This would explain why, after proNGF immunoprecipitation, the action of other proteins present in the CM (i.e., neuregulin-1) could be performed with less restrictions and full functional cell differentiation could be accomplished. This last assumption is strongly supported by findings reported by our group, demonstrating that the activation of neuregulin receptors (ErbB receptors) was necessary for the acquisition of the neuronal like-phenotype in PC12 cells. In that work we demonstrated that specific blocking of ErbB receptors, completely abolished neurite outgrowth in CM-treated PC12 cells. Blocking of ErbB receptors also abolished sodium currents induced by CM+k252a (García et al., 2013). Thus, considering these previous findings we can claim that neuregulin could be the main responsible for initiation of the cell differentiation and neuritogenesis, and that pro-NGF is involved in keeping that initial differentiation stage.

Additionally, neurite outgrowth, axonal specification and sodium currents, induced by depletion of proNGF from the CM (pNGFd-CM) could suggest a disruption of proNGF/sortilin/p75^NTR^ signaling complex. This is supported by our present finding, showing that the blockage of sortilin with NT counteracts proNGF actions, thus showing similar effects to the observed when proNGF is immunoprecipitated from the CM. Although, this does not preclude another possibility in which the immunoprecipitation might deplete several proNGF isoforms from the CM, which might have different functions. Additionally, the immunoprecipitation might bring down some proNGF interacting proteins, that might help to the proNGF to limit PC12 cell differentiation. Some studies have revealed inhibitory roles of pro-neurotrophins on differentiation and polarization, contrary to the effects of their mature counterparts. Such is the case for proBDNF, which inhibited neurite outgrowth in basal forebrain cholinergic neurons and reduced dendritic spine density in hippocampal neurons in a p75^NTR^ -dependent manner (Koshimizu et al., 2009). More recently, proBDNF was found to act as a potent collapsing factor for neurites in adult dorsal root ganglion neurons (DRG) neurons (Sun et al., 2012) and for growth cones in hippocampal neurons, in both cases through its binding to p75^NTR^ (Deinhardt et al., 2011). On the other hand, previous findings have demonstrated that a cleavage-resistant proNGF isoform promoted survival of mouse SCG neurons, increased the percentage of process-bearing neurons in culture (Matus et al., 1986; Fahnestock et al., 2004; Masoudi et al., 2009) and selectively promoted neurite outgrowth in a subset of NGF-responsive neurons, through a p75^NTR^-dependent mechanism (Howard et al., 2013).

Our current findings and previous reports involve the proNGF in pro-differentiation and anti-differentiation functions and are in agreement with the versatility in the cell response elicited by this proneurotrophin, in which the final outcome would depend on the relative levels of sortilin, TrkA and p75^NTR^ receptors (Nykjaer et al., 2004; Volosin et al., 2006, 2008; Arnett et al., 2007; Domeniconi et al., 2007; Jansen et al., 2007; Masoudi et al., 2009). Since the proNGF receptor, p75NTR, acts as a co-receptor for multiple partners, therefore, the presence of different co-receptors would permit the binding of different ligands, which can lead to diverse biological outcomes, such as apoptosis, survival (Lee et al., 2001; Teng et al., 2005; Schecterson and Bothwell, 2010), process retraction (Deinhardt et al., 2011), inhibition of axonal formation and sodium current induction (present work), and these different outcomes would depend upon the selective activation of distinct signaling mechanisms. For instance, stimulation of p75^NTR^ and Trk receptors can have opposite effects on neurite outgrowth, depending on the ligand, the levels of these receptors and the type of cell in which they coexist (Howard et al., 2013); for instance, the proNGF-p75^NTR^ interaction has been involved in promotion, as well as in inhibition of axonal growth (Bronfman and Fainzilber, 2004). Immunohistochemistry and electron microscopy revealed more prominent p75^NTR^ expression in axons than dendrites (Dougherty and Milner, 1999). Importantly, our present finding pointing to an inhibitory role of proNGF over axonal formation, was already suggested in previous work (Longart et al., 2009); this, together with the evidences showing the role of p75^NTR^, suggests a mechanism for axonal inhibition through this receptor and might explain in part our present results regarding the inhibition of axonal formation by proNGF. In addition and as explained above, it is also important to keep in mind the involvement of sortilin in the downregulation of the axonal development. The observation of the co-expression of the axonal and dendritic markers in neurites that would eventually become axons, is supported by previous findings, which have also shown the co-existence of the two markers in the neurites at pre-polarization stages, before the final axon specification (Matus et al., 1986; Pennypacker et al., 1991; Longart et al., 2009; Correa and Longart, 2010). Interestingly, in our previous work, the axonal final specification was reached when PC12 cells were treated with CM plus k252, which inhibits tyrosine kinase receptors (activated by NGF and other neurotrophins) and PKC; suggesting that other factors and signaling pathways are involved in the axonal specification and polarity processes (Longart et al., 2009; García et al., 2013).

To expand our understanding about the precise implication of proNGF over the limited capability of the CM to promote a complete neuronal-like differentiation in PC12 cells, it was important to demonstrate if reconstitution of pNGFd-CM with exogenous proNGF would modify its activity. It was known from previous studies that the CM, by itself, was not able to induce sodium currents in PC12 cells, unless proNGF was eliminated (Longart et al., 2009); at present, this is the only evidence that shows a negative regulation exerted by proNGF over sodium current induction. Thus, we first analyzed the effect of exogenous proNGF isoforms (wild type and mutated) over sodium current elicitation. The observation that these exogenous isoforms were able to induce sodium currents by themselves was unexpected; especially considering that depletion of endogenous isoforms from the CM induced sodium currents in PC12 cells. However, these results can explain why pNGFd-CM supplemented with pNGF*mut*, still induced sodium currents. Interestingly, the current density values of pNGF*mut* and pNGFd-CM +pNGF*mut* were almost identical, which could explain the sodium currents in the supplemented treatment. Now, more surprising were the results showing that treatments with pNGFd-CM supplemented with pNGF*wt*, induced larger sodium currents than in the pNGFd-CM treatment. One possible explanation could be that these larger sodium currents are the result of adding the sodium currents induced by pNGF*wt* plus the currents induced by the pNGFd-CM.

To further explain these results, it is important to mention that the concentrations of exogenous proNGF used here (10 and 100 ng/ml) were much higher than those normally found in biological settings, so this is probably not the cause why sodium currents still remained with those treatments. Other aspects to be considered are the structural differences between exogenous and endogenous isoforms. The exogenous isoforms have no post-translational modifications, since these are recombinant proteins expressed in bacteria, with a molecular mass of approximately 25 kDa (Alomone, Jerusalen, Israel). In contrast, the CM contains several proNGF isoforms ranging from 41–61 kDa (Longart et al., 2009) which can be suggestive of differentially glycosylated forms (Fahnestock et al., 2004) and might account for differences in their functions. Considering that mature NGF is capable to induce increments on sodium channel densities, neurite extension and axon formation in PC12 cells (Bouron et al., 1999; Longart et al., 2009) and that, pro-NGF can be processed by Furin (Seidah et al., 1996; Urban et al., 2013), thus generation of mature NGF from exogenous pNGF*wt* (through Furin), seems to be a possibility (unpublished results), to explain the persistence of sodium currents in PC12 cells. On the other hand, the mutated proNGF (pNGF*mut*) used in this study had point mutations that prevented cleavage by Furin; however, considering that this isoform was still able to induce sodium currents, we cannot rule out the possibility that pNGF*mut* could be processed by other enzymes to render the observed effect. Indeed, other cleavage sites used by different maturation enzymes have been reported, such as PACE-4, PC-2 (Pagadala et al., 2006), plasmine (Bruno and Cuello, 2006) and MMP-7 (Lee et al., 2001). Even though these results with the exogenous isoforms were unexpected, it is important to mention that to our knowledge there are not reports studying the effect of these isoforms over sodium currents, and this represent another important finding of our study.

Our results were also suggesting the possibility that the permanent binding of proNGF to sortilin could be involved in the downregulation of cell differentiation, polarization and sodium current induction, observed in PC12 cells treated with native CM. Considering this, previous reports have shown that the neuropeptide NT can inhibit proNGF activity, by competing with it for binding to the sortilin receptor (Nykjaer et al., 2004; Teng et al., 2005; Boules et al., 2006; Volosin et al., 2006; Domeniconi et al., 2007; Al-Shawi et al., 2008). Consequently, if sortilin receptor was involved in the proNGF-induced inhibitory pathway, it was likely that addition of NT to the CM could block proNGF activity. Precisely, treatment of PC12 cells with CM+NT, enhanced the percentage of differentiated cells, induced the presence of axons and sodium currents. These results strongly support the idea that proNGF, present in the CM, can be acting via sortilin, to downregulate PC12 cell differentiation, polarization and sodium current induction. In consequence, inhibition of proNGF action by blocking its interaction with sortilin (using NT), will allow other molecules in the CM to become available to induce cell differentiation, polarization and sodium current elicitation.

Further evaluations of our results improved the understanding on the intricacies of cell differentiation and polarization pathways regulated by proNGF. CM+NT treatment effects on cell differentiation were more discrete than the one observed with pNGFd-CM, for instance we did not observe an increase in the total neurite length with respect to the CM. The latter observations suggest that the affinity of NT for sortilin is probably lower than the affinity of proNGF for the p75^NTR^/sortilin complex in PC12 cells, at least in terms of total neurite elongation. On the other hand, it is unlikely that the cell responses to CM+NT were induced solely by the binding of NT to sortilin; since incubation of PC12 cells with DMEM+NT, did not affect cell differentiation, neurite length, axonal formation or sodium current induction. Therefore, we could infer that the sole occupation of sortilin with NT, did not allow a complete liberation of the inhibition exerted by proNGF in the CM, as the one observed with the pNGFd-CM treatment. This could be explained because; likely NT was not able to completely displace possible interactions between proNGF, p75^NTR^ and sortilin. Additionally, we cannot rule out the possibility that NT may be interfering with the binding of other molecules, present in the CM, to the sortilin receptor. For example, NT has also been shown to interfere with the binding of proBDNF to sortilin (Teng et al., 2005; Fauchais et al., 2008).

Interestingly, this work emphasizes and supports that proNGF present in the CM exerts an important negative regulation over key aspects of neuronal differentiation. Thus, it is feasible to suggest that this proneurotrophin is downregulating early signaling pathways that induce sodium channel activity and axon specification; exerted by its interaction with their natural receptors: TrkA or p75^NTR^- sortilin. The major findings of this work expand our knowledge about the functions initially described for proNGF, going from pro-apoptotic functions to regulation of neuronal differentiation. In conclusion, in this study we present evidences that support the role of proNGF as an inhibitory proneurotrophin present in the CM, which prevents neurite extension, axon specification and maturation in PC12 cells. In general terms, we can say that the limited capability of the CM over the neuronal-like differentiation of PC12 cells depends on the presence of proNGF released from sciatic nerves. In other words, immunodepletion of proNGF or inhibition of sortilin by addition of NT to the CM can be used to induce full and functional differentiation of PC12 cells, which might be further studied as a potential inductor of neuronal differentiation and regeneration.

## Author Contributions

AST, LG, CC, PF, RM and ML participated in the experimental work. RM, ML and CC participated in the experimental design. RM, ML, AST, LG and CC participated in the writing.

## Funding

This work was financially supported by Fundacion Instituto de Estudios Avanzados (IDEA) and the Venezuelan Ministry of Science.

## Conflict of Interest Statement

The authors declare that the research was conducted in the absence of any commercial or financial relationships that could be construed as a potential conflict of interest.

## Abbreviations

CM: sciatic nerve conditioned media
pNGFd-CM: sciatic nerve conditioned media immunodepleted of proNGF
NGF: nerve growth factor
ProNGF: precursor of nerve growth factor
*p*NGF*wt*: wild type proNGF
pNGF*mut*: mutated proNGF
PC12: cell line derived from a rat pheochromocytoma of adrenal medulla
TrK: tropomyosin-related kinase
TrkA: neurotrophin receptor A
p75^NTR^: p75 neurotrophin recptor
CNS: central nervous system
PKC: protein kinase C
PI3K: phosphatidylinositol-3-kinase
SCG: superior cervical ganglion neurons
DRG: dorsal root ganglion neurons
PPL: poly-L-lysine
%D: percentage of differentiated cells
DWN: differentiated without neurites
DLN: differentiated with long neurites
DSN: differentiated with short neurites
TLN: total length of neurites
PFA: paraformaldehyde
NGS: normal goat serum
k252a: generic inhibitor for TrkA, protein kinase C and other kinases
ERK1/2: extracellular-signal-regulated kinases 1/2
NT: neurotensin.

## References

[B1] Al-Shawi R. Hafner A. Olsen J. Olson J. Chun S. Raza S.. (2008). Neurotoxic and neurotrophic roles of proNGF and the receptor sortilin in the adult and ageing nervous system. Eur. J. Neurosci. 27, 2103–2114. 10.1111/j.1460-9568.2008.06152.x 18412630

[B2] Arnett M. G. Ryals J. M. Wright D. E. (2007). Pro-NGF, sortilin and p75NTR: potential mediators of injury-induced apoptosis in the mouse dorsal root ganglion. Brain Res. 1183, 32–42. 10.1016/j.brainres.2007.09.051 17964555 PMC2156563

[B3] Beattie M. S. Harrington A. W. Lee R. Kim J. Y. Boyce S. L. Longo F. M.. (2002). ProNGF induces p75-mediated death of oligodendrocytes following spinal cord injury. Neuron 36, 375–386. 10.1016/s0896-6273(02)01005-x 12408842 PMC2681189

[B4] Benfey M. Aguayo A. J. (1982). Extensive elongation of axons from rat brain into peripheral nerve grafts. Nature 296, 150–152. 10.1038/296150a0 7063015

[B5] Boules M. Fredrickson P. Richelson E. (2006). Bioactive analogs of neurotensin: focus on CNS effects. Peptides 27, 2523–2533. 10.1016/j.peptides.2005.12.018 16882457

[B6] Bouron A. Becker C. Porzig H. (1999). Functional expression of voltage-gated Na+ and Ca2+ channels during neuronal differentiation of PC12 cells with nerve growth factor or forskolin. Naunyn. Schmiedebergs Arch. Pharmacol. 359, 370–377. 10.1007/pl00005363 10498286

[B7] Bronfman F. C. Fainzilber M. (2004). Multi-tasking by the p75 neurotrophin receptor: sortilin things out? EMBO Rep. 5, 867–871. 10.1038/sj.embor.7400219 15470383 PMC1299130

[B8] Bruno M. A. Cuello A. C. (2006). Activity-dependent release of precursor nerve growth factor, conversion to mature nerve growth factor and its degradation by a protease cascade. Proc. Natl. Acad. Sci. U S A 103, 6735–6740. 10.1073/pnas.0510645103 16618925 PMC1458950

[B9] Castillo C. Carreño F. Villegas G. M. Villegas R. (2001). Ionic currents in PC12 cells differentiated into neuron-like cells by a cultured-sciatic nerve conditioned medium. Brain Res. 911, 181–192. 10.1016/s0006-8993(01)02683-x 11511389

[B10] Clewes O. Fahey M. S. Tyler S. J. Watson J. J. Seok H. Catania C.. (2008). Human ProNGF: biological effects and binding profiles at TrkA, P75NTR and sortilin. J. Neurochem. 107, 1124–1135. 10.1111/j.1471-4159.2008.05698.x 18808449

[B11] Correa G. Longart M. (2010). [Morphometric analysis of the differentiation process of hippocampal neurons *in vitro*]. Invest. Clin. 51, 501–518. 21365877

[B12] Deinhardt K. Kim T. Spellman D. S. Mains R. E. Eipper B. A. Neubert T. A.. (2011). Neuronal growth cone retraction relies on proneurotrophin receptor signaling through Rac. Sci. Signal. 4:ra82. 10.1126/scisignal.2002060 22155786 PMC3360552

[B13] Domeniconi M. Hempstead B. L. Chao M. V. (2007). Pro-NGF secreted by astrocytes promotes motor neuron cell death. Mol. Cell. Neurosci. 34, 271–279. 10.1016/j.mcn.2006.11.005 17188890 PMC2570110

[B130] Dougherty K. D. Milner T. A. (1999). p75^NTR^ immunoreactivity in the rat dentate gyrus is mostly within presynaptic profiles but is also found in some astrocytic and postsynaptic profiles. J. Comp. Neurol. 407, 77–91. 10.1002/(SICI)1096-9861(19990428)407:1<77::AID-CNE6>3.0.CO;2-S 10213189

[B14] Fahnestock M. Michalski B. Xu B. Coughlin M. D. (2001). The precursor pro-nerve growth factor is the predominant form of nerve growth factor in brain and is increased in Alzheimer’s disease. Mol. Cell. Neurosci. 18, 210–220. 10.1006/mcne.2001.1016 11520181

[B15] Fahnestock M. Yu G. Michalski B. Mathew S. Colquhoun A. Ross G. M.. (2004). The nerve growth factor precursor proNGF exhibits neurotrophic activity but is less active than mature nerve growth factor. J. Neurochem. 89, 581–592. 10.1111/j.1471-4159.2004.02360.x 15086515

[B16] Fauchais A.-L. Lalloué F. Lise M.-C. Boumediene A. Preud’homme J.-L. Vidal E.. (2008). Role of endogenous brain-derived neurotrophic factor and sortilin in B cell survival. J. Immunol. 181, 3027–3038. 10.4049/jimmunol.181.5.3027 18713973

[B17] García L. Castillo C. Carballo J. Rodríguez Y. Forsyth P. Medina R.. (2013). ErbB receptors and PKC regulate PC12 neuronal-like differentiation and sodium current elicitation. Neuroscience 236, 88–98. 10.1016/j.neuroscience.2013.01.026 23380500

[B18] Hamill O. P. Marty A. Neher E. Sakmann B. Sigworth F. J. (1981). Improved patch-clamp techniques for high-resolution current recording from cells and cell-free membrane patches. Pflugers. Arch. 391, 85–100. 10.1007/bf00656997 6270629

[B19] Howard L. Wyatt S. Nagappan G. Davies A. M. (2013). ProNGF promotes neurite growth from a subset of NGF-dependent neurons by a p75NTR-dependent mechanism. Development 140, 2108–2117. 10.1242/dev.085266 23633509 PMC3640218

[B20] Huang E. J. Reichardt L. F. (2001). Neurotrophins: roles in neuronal development and function. Annu. Rev. Neurosci. 24, 677–736. 10.1146/annurev.neuro.24.1.677 11520916 PMC2758233

[B21] Jansen P. Giehl K. Nyengaard J. R. Teng K. Lioubinski O. Sjoegaard S. S.. (2007). Roles for the pro-neurotrophin receptor sortilin in neuronal development, aging and brain injury. Nat. Neurosci. 10, 1449–1457. 10.1038/nn2000 17934455

[B22] Koshimizu H. Kiyosue K. Hara T. Hazama S. Suzuki S. Uegaki K.. (2009). Multiple functions of precursor BDNF to CNS neurons: negative regulation of neurite growth, spine formation and cell survival. Mol. Brain 2:27. 10.1186/1756-6606-2-27 19674479 PMC2743674

[B23] Lee F. S. Kim A. H. Khursigara G. Chao M. V. (2001). The uniqueness of being a neurotrophin receptor. Curr. Opin. Neurobiol. 11, 281–286. 10.1016/s0959-4388(00)00209-9 11399425

[B24] Longart M. García L. Castillo C. Martínez J. C. Medina R. Forsyth P.. (2009). Sciatic nerve conditioned medium depleted of pro-NGF modulates sodium currents and neurite outgrowth in PC12 cells. Neuroscience 159, 550–558. 10.1016/j.neuroscience.2008.12.063 19171180

[B25] Malavé C. Villegas G. M. Hernández M. Martínez J. C. Castillo C. Suárez de Mata Z.. (2003). Role of glypican-1 in the trophic activity on PC12 cells induced by cultured sciatic nerve conditioned medium: identification of a glypican-1-neuregulin complex. Brain Res. 983, 74–83. 10.1016/s0006-8993(03)03031-2 12914968

[B26] Masoudi R. Ioannou M. S. Coughlin M. D. Pagadala P. Neet K. E. Clewes O.. (2009). Biological activity of nerve growth factor precursor is dependent upon relative levels of its receptors. J. Biol. Chem. 284, 18424–18433. 10.1074/jbc.m109.007104 19389705 PMC2709390

[B27] Matus A. Bernhardt R. Bodmer R. Alaimo D. (1986). Microtubule-associated protein 2 and tubulin are differently distributed in the dendrites of developing neurons. Neuroscience 17, 371–389. 10.1016/0306-4522(86)90253-8 3517689

[B28] Nakamura K. Namekata K. Harada C. Harada T. (2007). Intracellular sortilin expression pattern regulates proNGF-induced naturally occurring cell death during development. Cell Death Differ. 14, 1552–1554. 10.1038/sj.cdd.4402173 17541425

[B29] Nykjaer A. Lee R. Teng K. K. Jansen P. Madsen P. Nielsen M. S.. (2004). Sortilin is essential for proNGF-induced neuronal cell death. Nature 427, 843–848. 10.1038/nature02319 14985763

[B30] Pagadala P. C. Dvorak L. A. Neet K. E. (2006). Construction of a mutated pro-nerve growth factor resistant to degradation and suitable for biophysical and cellular utilization. Proc. Natl. Acad. Sci. U S A 103, 17939–17943. 10.1073/pnas.0604139103 17093052 PMC1693851

[B31] Pennypacker K. Fischer I. Levitt P. (1991). Early *in vitro* genesis and differentiation of axons and dendrites by hippocampal neurons analyzed quantitatively with neurofilament-H and microtubule-associated protein 2 antibodies. Exp. Neurol. 111, 25–35. 10.1016/0014-4886(91)90047-g 1898595

[B32] Sandrock A. W. Matthew W. D. (1987). Substrate-bound nerve growth factor promotes neurite growth in peripheral nerve. Brain Res. 425, 360–363. 10.1016/0006-8993(87)90520-8 3427437

[B33] Schecterson L. C. Bothwell M. (2010). Neurotrophin receptors: old friends with new partners. Dev. Neurobiol. 70, 332–338. 10.1002/dneu.20767 20186712

[B34] Seidah N. G. Benjannet S. Pareek S. Savaria D. Hamelin J. Goulet B.. (1996). Cellular processing of the nerve growth factor precursor by the mammalian pro-protein convertases. Biochem. J. 314, 951–960. 10.1042/bj3140951 8615794 PMC1217149

[B35] Sun Y. Lim Y. Li F. Liu S. Lu J.-J. Haberberger R.. (2012). ProBDNF collapses neurite outgrowth of primary neurons by activating RhoA. PloS One 7:e35883. 10.1371/journal.pone.0035883 22558255 PMC3338794

[B36] Teng H. K. Teng K. K. Lee R. Wright S. Tevar S. Almeida R. D.. (2005). ProBDNF induces neuronal apoptosis via activation of a receptor complex of p75NTR and sortilin. J. Neurosci. 25, 5455–5463. 10.1523/jneurosci.5123-04.2005 15930396 PMC6724992

[B37] Urban D. Lorenz J. Meyborg H. Ghosh S. Kintscher U. Kaufmann J.. (2013). Proprotein convertase furin enhances survival and migration of vascular smooth muscle cells via processing of pro-nerve growth factor. J. Biochem. (Tokyo) 153, 197–207. 10.1093/jb/mvs137 23172302

[B38] Villegas G. M. Haustein A. T. Villegas R. (1995). Neuronal differentiation of PC12 and chick embryo ganglion cells induced by a sciatic nerve conditioned medium: characterization of the neurotrophic activity. Brain Res. 685, 77–90. 10.1016/0006-8993(95)00412-j 7583256

[B39] Villegas R. Villegas G. M. Longart M. Hernández M. Maqueira B. Buonanno A.. (2000). Neuregulin found in cultured-sciatic nerve conditioned medium causes neuronal differentiation of PC12 cells. Brain Res. 852, 305–318. 10.1016/s0006-8993(99)02109-5 10678757

[B40] Volosin M. Song W. Almeida R. D. Kaplan D. R. Hempstead B. L. Friedman W. J. (2006). Interaction of survival and death signaling in basal forebrain neurons: roles of neurotrophins and proneurotrophins. J. Neurosci. 26, 7756–7766. 10.1523/jneurosci.1560-06.2006 16855103 PMC6674285

[B41] Volosin M. Trotter C. Cragnolini A. Kenchappa R. S. Light M. Hempstead B. L.. (2008). Induction of proneurotrophins and activation of p75NTR-mediated apoptosis via neurotrophin receptor-interacting factor in hippocampal neurons after seizures. J. Neurosci. 28, 9870–9879. 10.1523/JNEUROSCI.2841-08.2008 18815271 PMC2578816

[B42] Wada A. (2006). Roles of voltage-dependent sodium channels in neuronal development, pain and neurodegeneration. J. Pharmacol. Sci. 102, 253–268. 10.1254/jphs.crj06012x 17072104

[B43] Xu X. Shrager P. (2005). Dependence of axon initial segment formation on Na+ channel expression. J. Neurosci. Res. 79, 428–441. 10.1002/jnr.20378 15635682

[B44] Zhou F.-Q. Zhou J. Dedhar S. Wu Y.-H. Snider W. D. (2004). NGF-induced axon growth is mediated by localized inactivation of GSK-3beta and functions of the microtubule plus end binding protein APC. Neuron 42, 897–912. 10.1016/j.neuron.2004.05.011 15207235

